# Opinion of German Immunologists on SARS-CoV-2: Results of an Online Survey

**DOI:** 10.7759/cureus.19393

**Published:** 2021-11-09

**Authors:** Harald Walach, Viviane Ruof, Raffaele Hellweg

**Affiliations:** 1 Research and Development, Change Health Science Institute, Berlin, DEU; 2 Breast Unit, University Hospital Heidelberg, Heidelberg, DEU

**Keywords:** immunology covid-19, scale validation, covid-19 vaccination, innate immune system, host, immune system, host factors, primary survey

## Abstract

Background

Little is known about the opinion of professional academic immunologists regarding the severe acute respiratory syndrome coronavirus 2 (SARS-CoV-2) pandemic.

Methodology

In this study, we designed an online survey to determine the opinion of immunologically competent academics on SARS-CoV-2 compared with seasonal flu (the infection fatality rate, infectivity, the challenge to the health system, the importance of vaccine development, and the importance of the virulence of the virus and host factors), in addition to collecting demographic status variables and information sources used. Links to the survey were sent to all German-speaking immunologists, bacteriologists, virologists, and infectiologists in Germany, Austria, and Switzerland.

Results

A total of 91 full datasets were returned after three waves of requests. Approximately half of the respondents were male and half were more junior. Slightly more than half of the respondents said that the infection fatality rate and the infectivity were higher compared to flu, and 82% said that the challenge to the health system is higher. Overall, 52% found that the immune system is more important than the virus, and a majority (59%) supported the current practice of vaccination development by telescoping. A majority were of the view that conspiracy theories and non-pharmacological interventions pose a greater danger than the virus. Respondents who were more junior but well-published and mostly informed by public channels were more likely to support a mainstream view.

Conclusions

German-speaking immunological professionals hold widely diverging opinions regarding SARS-CoV-2. Over half of the surveyed professionals considered SARS-CoV-2 to be more dangerous and infective than the seasonal flu. However, the majority considered the health system to be under higher strain. Interestingly, more than half of them found host factors more important.

## Introduction

During the severe acute respiratory syndrome coronavirus 2 (SARS-CoV-2) pandemic, virologists dominated the public view in print and television media, at least in Germany. Immunologists were rarely heard or seen in the public domain. In the scientific literature, there is scarce information regarding the opinions of immunologists about this pandemic, apart from reviews on immunological findings [1-7]. As findings accrue that 34% up to 81% of previously unaffected individuals might have preexisting immunity due to T-cells responsive to coronaviruses in general through cross-reactivity [1,2,8], we thought it interesting to understand the opinions of immunologists and other specialists, such as virologists, bacteriologists, and infectiologists, on infection. Here, we report the results of an online survey directed at all immunologists, infectiologists, bacteriologists, and virologists in university centers in Germany, Austria, and Switzerland.

## Materials and methods

We used publicly available information (university affiliations, names, and email addresses of immunologists) to find names and email addresses for all immunologists, bacteriologists, virologists, and infectiologists active in German, Austrian, and Swiss universities. The search was conducted manually by a medically trained person (RH) and yielded 1,025 individuals for whom email addresses could be procured. A survey using the German online survey tool Social Science Survey (SocSciSurv) (SoSci Survey GmbH, Munich, Germany) was developed, following a predefined protocol that was approved by the institutional ethics committee (University of Witten/Herdecke, S-20/2021). The Social Science Survey Tool is a professional survey platform that controls IP addresses such that double entries from the same address are prevented. It logs the time spent on the questionnaire and implements quality controls that allow distinguishing between seriously filled-in surveys and rapid scrollers.

The questionnaire and the survey invitation are presented in a translated version in the Appendices. The questionnaire contained the following questions and related response categories (Table 1).

**Table 1:** Survey questions. *“Habilitation” is a qualification typical for some European countries (such as Germany, Austria, Switzerland, France, Italy, Poland, and others) that requires another lengthy thesis or a larger research portfolio than a PhD thesis, involves a faculty examination, and is the requirement for being appointed professor or being able to supervise PhD students on a formal basis. SARS-CoV-2: severe acute respiratory syndrome coronavirus 2

Question number	Question	Response categories
1	Age	Open
2	Gender	Male/female/diverse
3	Academic education/position	PhD, habilitation*, professor
4	Research experience in years	<5 years, <10 years, <20 years, <40 years, >40 years
5	Number of publications in categories	<20, <50, <100, <200, <300, >300
6	The severity of SARS-CoV-2 in comparison with seasonal flu is … in terms of…	Lower, similar, higher, clearly higher
6A	Infection fatality rate	Lower, similar, higher, clearly higher
6B	Infectivity	Lower, similar, higher, clearly higher
6C	Challenge to the health system	Lower, similar, higher, clearly higher
7	The public threat is mainly…	By the virus, non-pharmaceutical interventions, or conspiracy theories
8	Vaccination development … should be	By telescoping, should be normal, no vaccine necessary
9	More important is the virulence of the virus or host factors (immune system)	
10	In your opinion, what are the major information sources of the public	Public television, print media, alternative sources
11	What are the major information sources of the respondent	Public channels, scientific information, own analysis of figures and data, exchange with colleagues
12	Option for free text answer	
13	Voluntary contact details if interested in an interview	

The questionnaire was mounted on SocSciSurv and sent out in three waves starting April 23, 2021 (beginning of the first wave), May 26, 2021 (beginning of the second wave), and June 14, 2021 (beginning of the third wave), with the last dataset arriving on June 16, 2021). The cover text included the message to only fill in the questionnaire if a respondent was active in the field of immunology, clinical, or research. IP addresses were not revealed. Thus, the survey was strictly anonymous.

## Results

The survey was accessed by 94 individuals, with three surveys having missing answers for all questions. These surveys were removed to give a final sample of 91 complete surveys. The description of the respondents is presented in Table 2, and the results of the survey are presented in Table 3.

**Table 2:** Description of the respondents: German-speaking academic immunologists from Germany, Switzerland, and Austria. *“diverse” was part of the answer option, but not chosen; “missing data” are true missing data.

Variable/Question (n = 91)	Number/Mean (standard deviation)	Percentage
Answers in wave		
1	59	64.8%
2	13	14.3%
3	19	20.9%
Gender		
Male	52	57.1%
Female	31	34.8%
Missing*	8	8.8%
Status		
PhD	39	42.9%
Habilitation	8	8.8%
Professor	35	38.5%
Missing	9	9.9%
Research experience		
<5 years	16	17.6%
<10 years	12	13.2%
<20 years	23	25.3%
<40 years	31	34.1%
>40 years	1	1.1%
Missing	8	8.8%
Number of publications		
<20	25	27.5%
<50	19	20.9%
<100	15	16.5%
<200	12	13.2%
<300	7	7.7%
>300	2	2.2%
Missing	11	12.1%
Age (12 missing)	47.9 (11.5)	

**Table 3:** Result of the survey: immunologists’ opinions presented as numbers and percentages. *These items were coded “yes” and “no”; only the “yes” answers and the missing data are presented, and the “no” answers comprise the rest. NPI: non-pharmaceutical intervention; TV: television

	Number	Percent
Infection Fatality Rate compared with seasonal flu is …		
lower	1	1,1
similar	13	14,3
higher	43	47,2
clearly higher	26	28,6
missing	8	8,8
Infectivity compared with seasonal flu is …		
lower	2	2,2
similar	25	27,5
higher	40	44,0
clearly higher	14	15,4
missing	10	11,0
The challenge to the health system is …		
similar	6	6,6
higher	30	33,0
clearly higher	45	49,4
missing	10	11,0
More important is		
the virus	32	35,2
the immune system	48	52,7
missing	11	12,1
The highest danger poses		
the virus	34	37,4
NPIs	19	20,9
Conspiracy theories	29	31,9
missing	9	9,9
Vaccination development should proceed		
by telescoping	54	59,3
as normal	24	26,4
Vaccine unnecessary	4	4,4
missing	9	9,9
*Information source of the public is mainly….		
TV and public radio	70	76,9
missing	3	3,3
Print media	24	26,4
missing	3	3,3
Alternative media (internet media)	32	35,2
missing	3	3,3
*My main information source is		
Public channels (TV, print media, public radio)	61	67,0
missing	3	3,3
Scientific information	76	83,5
Missing	3	3,3
Own analysis of data and figures	56	61,5
Missing	3	3,3
Exchange with colleagues	68	74,7
missing	3	3,3

We correlated the variables that were descriptive of the sample, with the variables denoting opinions, using Spearman’s rank correlation. There were only two significant (p < 0.05) groups of correlations: immunologists thought that when the public was using print media as their main source of information, the higher their status was (r = 0.22), the more experience they had (r = 0.33), and the more publications they had (r = 0.28). The more publications they had themselves, the less they informed themselves via scientific literature (r = 0.29). As this was a purely exploratory analysis, no corrections for multiple testing were applied to this set of 19 variables correlating with each other.

We created an ad-hoc scale by aggregating variables. We used all variables denoting an opinion on SARS-CoV-2 and calculated a single scale score. We did not omit any variables or distort data to avoid selective reporting and multiple testing. This scale described how much a respondent subscribed to the general mainstream opinion about the SARS-CoV-2 pandemic. To create this scale, we summed the answers to the items asking about the infection fatality rate, the infectivity, the challenge to the health system, the importance of the virus versus immune system, the danger posed by the virus versus non-pharmaceutical interventions (NPIs), and the support for vaccination development by telescoping. For convenience, we call this the “mainstream score.” We assumed that someone had an opinion close to the mainstream view if they said that the “infection fatality rate was clearly higher than the flu,” “infectivity was clearly higher than the flu,” “the challenge to the health system was much higher than with the flu,” “the virus was more important than the immune system,” “the danger posed by the virus was greater than that by NPIs,” and that there was a “necessity for vaccinations and for telescoping vaccine development.” The variables were scored such that a higher score in an item would yield a higher mainstream score. For instance, if the infection fatality rate, the infectivity, and the challenge to the health system were rated “clearly higher,” it would yield a score of 4 for each variable, that is, a score of 12 for a person rating all three items as “clearly higher.” The other items were recoded as dummy variables, such that the positive answer to those items would yield a score of 1. Thus, we created a new scale with a theoretical range from 4, denoting an opinion clearly diverging from the mainstream opinion, to 18, denoting an opinion completely supporting the mainstream narrative represented by these items.

**Figure 1:**
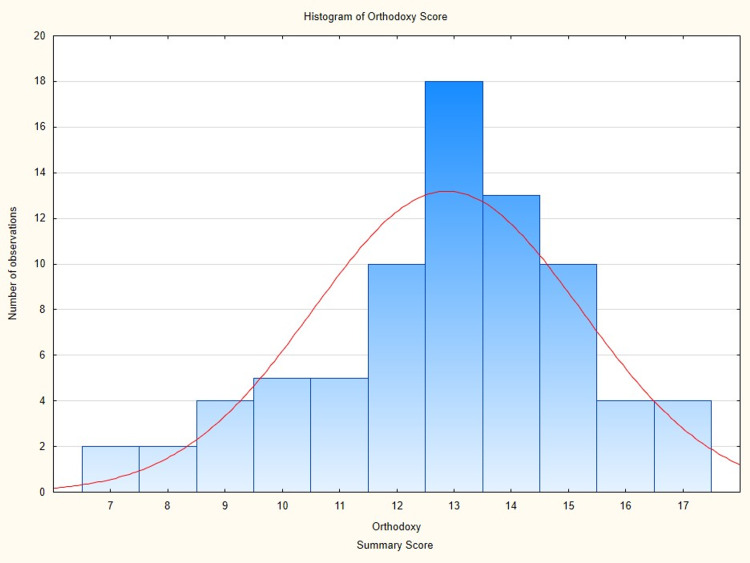
Histogram of “mainstream” summary score.

This ad-hoc scale showed reasonable consistency after conducting a reliability analysis (Cronbach’s alpha standardized = 0.74, mean item intercorrelation = 0.26) and was approximately normally distributed (Figure 1).

The mean score was 13.6 (standard deviation = 2.5), and it ranged from 7 to 17 (theoretical range = 4 to 18). A principal component analysis confirmed that the scale was unidimensional.

We used a regression model to clarify the variance in the “mainstream score” by status variables and information sources using a stepwise multiple regression model. This produced a significant regression equation which is presented in Table 3.

**Table 4:** Regression on the mainstream score. R^2^_adj_ = 0.13, F _4/66_ = 3.5, p = 0.01 (intercept calculated but not presented).

Variable	Beta-weight (standard error)	t-score	P-value
Information by public channels	0.25 (0.12)	2.18	0.03
Number of publications	0.48 (0.18)	2.6	0.01
Status	−0.40 (0.19)	−2.13	0.04
Information by own analysis	−0.21 (0.12)	−1.85	0.07

This regression equation could predict 13% of the variance in the “mainstream score.” Immunologists tended toward an opinion more closely confirming to the “mainstream opinion” (holding the opinion that SARS-CoV-2 has a higher infection fatality rate than flu, is more infectious, challenges the system more, the virus is more important than the immune system, the true danger stems from the virus, and that vaccinations are necessary and should be telescoped) if they informed themselves via public channels, had a higher number of publications, but lower status, and performed less of their own analysis of data and figures.

## Discussion

It is interesting to note that among German-speaking immunologists from Germany, Switzerland, and Austria there is a larger variance of opinion than might be expected. The basic description shows that we have captured roughly half of junior and senior staff. Overall, 15-30% of surveyed individuals held the opinion that the pandemic is roughly comparable with a severe influenza epidemic, whereas most agreed that the challenge to the health system is higher with the SARS-CoV-2 pandemic. However, more than half of our respondents thought that the immune system is more important than the virus itself. A majority were of the opinion that either the NPIs themselves (21%) or conspiracy theories (32%) are more dangerous than the virus. Moreover, 60% supported the telescoped development of vaccines.

The psychometric analysis of the opinion items supported a “mainstream” score that reflects what we would call the mainstream narrative. This score nearly covered the full range; the lowest participant scored 7, where the theoretically lowest score is 4, and the person with the most orthodox opinion scored 17, where the theoretical maximum is 18. This score was normally distributed, with the median (13) and mean (13.6) being similar. Moreover, junior immunologists appeared to be very active in publishing, informed themselves via public channels, and did not conduct their own analyses that support such an opinion. This ad-hoc scale was psychometrically sound, with reasonable internal consistency, and was unidimensional. Hence, using it as an aggregate variable is justified.

One might challenge our operationalization of what is “mainstream.” We submit that it was an ad-hoc common-sense decision to denote as “mainstream” the opinion that SARS-CoV-2 is associated with a higher infection fatality rate than flu, is more infectious and challenging to the healthcare system than flu, that vaccine development should proceed by telescoping, and that the virulence of the virus is more important than host factors. However, these appear to be at least some of the most important elements that comprise the mainstream narrative, if not all of them, and thus it is justified in our view to take these elements of an opinion as reflecting a mainstream narrative. The psychometric analysis supports this decision.

It is tempting to speculate why more junior, well-published immunologists seem to hold a view more closely aligned with the mainstream view. The fact that information from public channels is the main source of information for those holding a view closer to the mainstream opinion can help understand this: younger academics who are eager to climb the career ladder are likely more pressurized to finalize their own projects and will thus concentrate on their own topics and inform themselves about other issues, such as SARS-CoV-2, if it is not at the center of their interest, via mainstream channels. Another potential interpretation is that younger academics have to be, by necessity, more aligned with what they perceive to be mainstream in order to not jeopardize their career.

These findings have to be viewed against the obvious limitations: we reached only a small fraction (9%) of all potential respondents. This is likely because normal activities were rather disrupted by the pandemic or a higher job demand made the idea of participating in a survey unattractive. Experiences with other surveys, however, show that even small percentages of participation reflect prevalent opinions. In a representative survey of psychotherapists, we conducted a non-responder analysis and observed that there was no difference between responders and non-responders in core opinions [9]. Other recent studies show that there is no significant difference in opinions or status between non-responders and responders [10-12]. While it is unclear whether we can transfer this finding to other groups and times, it is certainly reassuring to a degree. The survey system was professional and prevented double entries via IP controls.

Our data are only a glimpse into the opinion of a profession that is important in the pandemic. We see that the opinions are far from uniform, with some variation. Our construction of a “mainstream” score was quite successful and demonstrated a near perfect normal distribution with the majority situated somewhere in the middle.

## Conclusions

The opinions of immunologically competent academics on the pandemic vary widely. Approximately one-third of immunologists view the SARS-CoV-2 pandemic to be similar to severe flu, but most agree that the challenge to the health system is higher in this pandemic than during flu outbreaks. The more seasoned and senior immunologists tend toward a view regarding the pandemic which deviates more from the mainstream opinion. More than half emphasize what has been neglected so far: the importance of the immune system, or host factors, and the potential dangers of NPIs, as well as that of conspiracy theories. Perhaps it might be worthwhile to scrutinize the professional opinions of this group more carefully in interviews and qualitative studies. We have successfully constructed an “orthodoxy score.” More junior, well-published immunologists who inform themselves mainly via public channels and not their own analysis of the literature tend to hold a more orthodox view.

## Appendices

Text of the survey invitation (translated; original by author) and the survey itself

This is an anonymous survey in German that is directed at immunologists and immunologically trained medical specialists in university hospitals and hospitals in Germany, Austria, and Switzerland. We want to capture the professional opinion of this group of specialists regarding the COVID-19 pandemic and topics related to it.

The survey contains 12 simple questions and can be answered in maximally 15 minutes.

Please answer this survey only if you have a medical or health science basic academic training, if you hold an MD or PhD degree, and if you are a specialist in any field of immunology, either as a researcher or in clinical practice. The link to this survey is for you only and should not be passed on, as we are inviting those in question directly.

The survey is strictly anonymous. We are saving person-specific data (IP addresses, email addresses) only for the time necessary to do this survey. The goal of the survey is exclusively to increase scientific knowledge. Thus, it is important that you answer these questions according to your professional expertise truthfully.

By clicking the link you are consenting to participate in the survey and allowing us to analyze your data anonymously. If you do not want to participate in the survey, simply do not continue or press “Exit.”

At the end of the survey, you can add free text, if something is important for you, or you can enter your contact details (email, phone number) if you wish to participate in a phone interview. We will use those exclusively for this purpose and delete the information after the survey is finished.

This study is organized by Prof. Harald Walach, who is also responsible for data protection and following good scientific practice.

**Table 5:** Survey questions. SARS-CoV-2: severe acute respiratory syndrome coronavirus 2; TV: television

Age			
Gender	□	Male	
	□	Female	
	□	Diverse	
Status	□	PhD/MD	
	□	Habilitation	
	□	Professor or similar position	
Research experience in years			
	□	<5	
	□	<10	
	□	<20	
	□	<40	
	□	>40	
Own publications as author or coauthor in the peer-reviewed literature			
	□	<20	
	□	<100	
	□	<200	
	□	<300	
	□	>300	
How do you see the severity of the SARS-CoV-2 pandemic compared to seasonal influenza in terms of			
	a) Infection fatality rate	□	Lower
		□	About equal
		□	Higher
		□	Clearly higher
	b) Infectivity	□	Lower
		□	About equal
		□	Higher
		□	Clearly higher
	c) Challenge for the health system	□	Lower
		□	About equal
		□	Higher
		□	Clearly higher
What poses the main threat	□	The virus	
	□	The political interventions (“non-pharmacological interventions”)	
	□	Alternative theories (“conspiracy theories”)	
Development of vaccines			
	□	Development of new vaccines without prior safety tests according to traditional EMA guidelines (“telescoping” of phases) is justified and important	
	□	Development should follow the traditional trajectory, even if it takes longer	
	□	We do not need vaccines	
The decisive factor is	□	The virulence of the virus	
	□	The potency of the immune system	
In my opinion the majority of the population are informing themselves via … (multiple options possible)	□	Public radio news and TV-channels	
	□	Newspapers and magazines	
	□	Alternative internet media	
I get my information via	□	Public channels (radio, TV, newspapers)	
	□	Scientific original publications	
	□	Own analysis of public data (numbers of diseased patients and fatalities from public statistical sources)	
	□	Exchange with colleagues, opinions of other colleagues or scientific societies	
If you are interested in participating in an interview please leave your email address and/or phone number			
Thank you for taking the time to answer the survey! If you are interested in participating in an interview please leave your email address and/or phone number			
